# Involvement of necroptosis in the selective toxicity of the natural compound (±) gossypol on squamous skin cancer cells in vitro

**DOI:** 10.1007/s00204-023-03516-1

**Published:** 2023-05-21

**Authors:** Lisa Haasler, Claudia von Montfort, Arun Kumar Kondadi, Mathias Golombek, Lara Ebbert, Chantal-Kristin Wenzel, Wilhelm Stahl, Andreas S. Reichert, Peter Brenneisen

**Affiliations:** Institute of Biochemistry and Molecular Biology I, Medical Faculty, Heinrich-Heine University Düsseldorf, 40225 Düsseldorf, Germany

**Keywords:** Cutaneous squamous cell carcinoma, Gossypol, Mitochondrial dysfunction, Necroptosis, Keratinocytes

## Abstract

Cutaneous basal and squamous cell carcinoma reflect the first and second most common type of non-melanoma skin cancer, respectively. Especially cutaneous squamous cell carcinoma has the tendency to metastasize, finally resulting in a rather poor prognosis. Therapeutic options comprise surgery, radiation therapy, and a systemic or targeted chemotherapy. There are some good treatment results, but overall, the response rate of newly developed drugs is still modest. Drug repurposing represents an alternative approach where already available and clinically approved substances are used, which originally intended for other clinical benefits. In this context, we tested the effect of the naturally occurring polyphenolic aldehyde (±) gossypol with concentrations between 1 and 5 µM on the invasive squamous cell carcinoma cell line SCL-1 and normal human epidermal keratinocytes. Gossypol treatment up to 96 h resulted in a selective cytotoxicity of SCL-1 cells (IC_50_: 1.7 µM, 96 h) compared with normal keratinocytes (IC_50_: ≥ 5.4 µM, 96 h) which is mediated by mitochondrial dysfunction and finally leading to necroptotic cell death. Taken together, gossypol shows a high potential as an alternative anticancer drug for the treatment of cutaneous squamous cell carcinoma.

## Introduction

Skin cancer, mainly the out-of-control growth of specific cells in the epidermis, roughly comprises cutaneous melanoma (CM) and non-melanoma skin cancer (NMSC). NMSC are subdivided in the two keratinocyte skin cancers (KSC) named basal cell carcinoma (BCC) and cutaneous squamous cell carcinoma (cSCC), reflecting the first and second most common types of skin cancer (Dubas and Ingraffea 2013; Fania et al. 2021). Non-melanoma skin cancer has been demonstrated to associate with significant morbidity, mortality, and economic burden (Cakir et al. 2012; Nehal and Bichakjian 2018). The Global Burden of Disease Study (GBDS 2019) (Fitzmaurice et al. 2019) pointed out NMSC is the topmost of the global top ten cancers in 2017 with 7.7 million new cases of NMSC of which roughly 25% represent squamous cell carcinomas. While both BCC and cSCC are normally described to exhibit a benign clinical behavior, about 5% of cSCC cases will become locally advanced, recur or metastasize (Ribero et al. 2017; Varra et al. 2018). Among other risk factors, UVB irradiation (280–320 nm) is associated with the highest risk for the development of BCC and cSCC (Watson et al. 2016; Laikova et al. 2019). Options of treatment against cSCC comprise surgery, radiation therapy, and a systemic or targeted chemotherapy, either each of those alone or in a combination (Burton et al. 2016; Dessinioti and Stratigos 2022). Patients with advanced unresectable or metastatic cSCC have access to a systemic treatment with platinum compounds, 5-fluorouracil, methotrexate, taxanes, and anthracyclines (Nakamura et al. 2013; Ribero et al. 2017) or a more targeted treatment with small molecule drugs or monoclonal antibodies targeting specific proteins (Corchado-Cobos et al. 2020; García-Foncillas et al. 2022). In a number of clinical trials, it has become evident that the overall response rate is rather modest and accompanied by resistances (Wheeler et al. 2008) as well as side effects from skin reactions to anaphylaxis (Hu et al. 2007; Marti et al. 2016; Agirgol et al. 2020). An alternative to the high expenditure of time and exorbitant rising costs for the development of novel drugs is the modified use of already available and clinically approved substances, which originally intended for other clinical benefit (drug repurposing) (Gupta et al. 2013; Zhang et al. 2020). As an example, the chlorinated 4-aminoquinoline derivative chloroquine originally developed for prophylaxis and treatment of malaria was repurposed as inhibitor of autophagy and anticancer agent (Vlahopoulos et al. 2014). Further on, (±) gossypol (GP), a naturally occurring polyphenolic aldehyde from cottonseed (Adams et al. 1960) and originally being tested as a male contraceptive (Coutinho 2002), is now in the focus as an anticancer drug (Zeng et al. 2019; Liu et al. 2022). As a BH3 mimetic agent, it was shown that GP inhibited the anti-apoptotic proteins BCL-2, BCL-xL, and MCL-1 (Opydo-Chanek et al. 2017; Melo et al. 2022). Recently, it was described that GP acetate at high concentrations led to an autophagic block in the human non-small-cell lung cancer cell line A549 (Cai et al. 2022). Previously, we demonstrated that GP exhibited a selective toxicity on A375 melanoma cells via mitochondrial dysfunction finally resulting in apoptotic cell death (Haasler et al. 2021). In this in vitro study, we focused on the effect of GP on the NMSC cell line SCL-1 which is a model of cSCC showing malignant growth characteristics and an invasive capacity in vivo (Boukamp et al. 1982). GP caused a selective toxicity in the tumor cells compared to normal keratinocytes which was mediated by mitochondrial changes and finally resulted in necroptotic cell death.

## Materials and methods

### Materials

The used chemicals and Dulbecco’s modified Eagle’s medium (DMEM) were purchased from Sigma-Aldrich (Taufkirchen, Germany) or Merck (Darmstadt, Germany), if not otherwise stated. ABT-199/venetoclax (CAS 1257044-40-) and (±) gossypol (GP, CAS 303-45-7) were obtained from Abcam (Cambrigde, UK). Fetal bovine serum (FBS) was from Pan-Biotech (Aidenbach, Germany). Penicillin/Streptomycin was purchased from Biochrom (Berlin, Germany) and Glutamax from Gibco (Darmstadt, Germany). The keratinocyte basal (C-20211) and growth medium including SupplementMix (C-20011) were obtained from PromoCell (Heidelberg, Germany). The pan-caspase inhibitor Z-VAD(OMe)-FMK (zVAD) was purchased from Santa Cruz Biotechnology (Heidelberg, Germany). Tetramethylrhodamine methylester (TMRM) and Molecular Probes MitoTRACKER™ Green FM were obtained from Thermo Fisher Scientific (Waltham, Massachusetts, USA). The Seahorse XF Cell Mito Stress Test Kit (Cat. 103015-100) was obtained from Agilent Technologies (Waldbronn, Germany). The ProLong Gold Antifade Reagent with DAPI (Cat. 8961) was ordered from Cell Signaling Technology (Danvers, Massachusetts, USA). Caspase 3/7 (Cat. 22795), Caspase 8 (Cat. 22812), and Caspase 9 (Cat. 22813) Activity Apoptosis Assays were purchased from AAT Bioquest (Biomol, Hamburg, Germany). The lactate dehydrogenase (LDH) ELISA kit (Cat. ab183367) was from Abcam and the DC™ protein assay kit was purchased from Bio-Rad (Feldkirchen, Germany).

The following primary antibodies were used: polyclonal anti-PARP (Cat. 9542), the monoclonals anti-Bax (Cat. 5023), anti-BcL-xL (Cat. 2764), anti-phospho-RIP3 (Ser227; Cat. 93654), and anti-ß-Tubulin (Cat. 2128) from Cell Signaling Technology; monoclonal anti-Bcl-2 (Cat. Ab32124) from Abcam; monoclonal anti-RIP3 (Cat. sc-374639) from Santa Cruz Biotechnology. As secondary antibodies, the horseradish peroxidase (HRP)-conjugated goat anti-rabbit IgG (Cat. 111-035-144) from Dianova (Hamburg, Germany) and Alexa Fluor 568 goat anti-mouse IgG (red; Cat. A-11004) from Thermo Fisher were used.

### Cell culture

The human cutaneous squamous carcinoma cell line SCL-1 was a gift from Prof. Dr. Norbert Fusenig from the German Cancer Research Center (DKFZ, Heidelberg, Germany) (Boukamp et al. 1982). The human melanoma cell line A375 (ATCC CRL-1619) was obtained from the American Type Culture Collection (ATCC, Virginia, USA). Normal human epidermal keratinocytes (NHEK, C-12001) were ordered from PromoCell (Heidelberg, Germany). SCL-1 and A375 tumor cells were cultured in Dulbecco’s modified Eagle’s medium (DMEM, low glucose), supplemented with 10% fetal bovine serum (FBS), streptomycin (100 µg/ml), penicillin (100 U/ml), and GlutaMAX™ (2 mM) at 37 °C in 5% CO_2_. NHEK were cultured in keratinocyte growth medium (C-20011, PromoCell) including SupplementMix (C-39016), streptomycin (100 µg/ml) and penicillin (100 U/ml) at 37 °C in 5% CO_2_. Subconfluent cells (70–80% confluence) were used for all experiments, if not otherwise stated. For treatment, SCL-1 and A375 were incubated in high glucose (4500 mg/L) DMEM without FBS, while NHEK were grown in keratinocyte basal medium (C-20211, PromoCell). Gossypol and other substances such as ABT-199, inhibitors, and fluorescent dyes were directly added to the cells at the appropriate concentrations.

### Cell viability assay

The cell viability was either measured by the MTT (3-(4,5-dimethylthiazol-2-yl)-2,5-diphenyltetrazolium bromide) or sulforhodamine B (SRB) assay which are based on the activity of mitochondrial dehydrogenases (Mosmann 1983) and on the pH-dependent staining of total proteins, respectively (Maydt et al. 2013). For MTT assay, the enzyme catalyzes the conversion from MTT to a purple formazan dye. Subconfluent cells were treated with different concentrations of GP or mock-treated in 24-well plates. After washing with PBS, MTT solution (0.5 mg/ml) was directly added to the cells and incubated between 0.5 and 2 h depending on the cell type. After removal of MTT, the cells were washed with PBS and 500 µl DMSO per well was added for formazan extraction. Absorbance was measured at 570 nm with a plate reader (FLUOstar OPTIMA, BMG Labtech, Ortenberg, Germany). The mock-treated control was set at 100%. The SRB assay was performed with subconfluent cells in four-well plates. Cells were treated with different concentration of GP or mock treated. Subsequently, cells were washed with PBS and fixed with 10% (w/v) cold trichloroacetic acid solution (500 µl/well) for 1 h at 4 °C. After washing several times with dH_2_O, cells were dried at RT. For the staining, cells were incubated with SRB solution (0.4% (w/v) in 1% acetic acid, 300 µl/well) for 15 min at RT, washed with 1% acetic acid and dried at RT. For extraction of SRB, 400 µl TRIS-Base (10 mmol/l) was added per well and the plate was gently rotated for 5 min. The absorbance was measured at 492 nm minus background values at 620 nm using a microplate reader (Tecan M200 pro, Männedorf, Switzerland). Cell viability of mock-treated cells was set at 100%.

### Intracellular measurement of GP (HPLC)

Cellular uptake of GP was determined by high performance liquid chromatography (HPLC) after treatment of cells with 5 μM GP. Tumor and normal cells were grown to subconfluence in Ø 10 cm culture dishes. After incubation with GP for different time periods and washing with PBS, cells were harvested in 2 ml PBS. Samples were centrifuged at 500×g for 8 min at 4 °C, washed with PBS, and centrifuged at 5.000xg for 6 min at 4 °C. Cell pellets were resuspended in 150 μl acetonitrile (AcN), mixed thoroughly, and centrifuged at 20.000xg for 5 min at 4 °C. For analysis, 50 μl of the AcN extract was injected, and the GP concentration was quantified using a standard curve. HPLC was performed on a Supelco pKb 100 (250 × 4.6 mm) column with a mobile phase consisting of AcN/water/trifluoroacetic acid (90/10/0.1, v/v/v) at a flow rate of 1.0 ml/min (0–10 min) and UV detection at 367 nm. Retention time of GP was around 5 min (Haasler et al. 2021). Only intracellular GP and not its metabolites were measured. For protein quantification, the solvent residue was evaporated and the cell pellet was solved in 1% SDS lysis buffer containing 0.1% protease inhibitor cocktail and sonicated. For quantification of the intracellular GP content, the concentration of GP was set in relation to the protein amount, which was calculated using the DC™ Protein Assay Kit.

### Mitochondrial membrane potential (ΔΨm)

To study changes in ΔΨm, SCL-1 tumor cells and NHEK were seeded on glass bottom dishes (Ø 3.5 cm, MatTek, Son, Netherlands) and incubated with 2.5 μM GP or mock treated (DMSO) for 2 and 4 h, respectively, or with 10 μM carbonyl cyanide m-chlorophenyl hydrazone (CCCP), an oxidative phosphorylation uncoupler (Mahalaxmi et al. 2022), for 2 h as positive control. After incubation, cells were washed with PBS and loaded with 100 nM of the mitochondrial membrane potential sensitive dye tetramethylrhodamine methyl ester (TMRM) (Creed and McKenzie 2019) and 100 nM of MitoTRACKER™ Green for 0.5 h at 37 °C. After washing with PBS, fresh medium was added to the cells. Cells were analyzed with an Ultraview spinning disc confocal microscope (PerkinElmer Corporation, Waltham, Massachusetts, USA). At least 20 image stacks per sample were evaluated. The ΔΨm was calculated by the quotient TMRM to MitoTRACKER™ Green. The mock-treated controls were set at 100%.

### Mitochondrial fragmentation

To test for mitochondrial fragmentation as a parameter of mitochondrial dysfunction, the squamous tumor cells and normal keratinocytes were treated as described above for ΔΨm. MitoTRACKER™ Green images were used to evaluate mitochondrial morphology and the quantification was performed as described before (Duvezin-Caubet et al. 2006; Aplak et al. 2020): tubular, at least one mitochondrial tubule of 5 μm or more; intermediate, at least one tubule between 0.5 and 5 μm; fragmented, no tubules of more than 0.5 μm in length. At least 30 cells per sample were analyzed.

### Cell characterization and Mito Stress Test

To assess mitochondrial function in cells, the Seahorse XF Cell Mito Stress Test was performed according to the manufacturer’s protocol based on the measurement of the oxygen consumption rate (OCR) (Gu et al. 2021) including parameters such as basal and maximal respiration, spare respiratory capacity (SRC), ATP production, and proton leak. A characterization of each cell type was performed to determine the most suitable conditions for the experiments. For that, different cell numbers for each cell type were seeded in a Seahorse 96-well plate (80 µl/well). Different concentrations of the inhibitor oligomycin (Oligo), the protonophore carbonyl cyanide p-trifluoro-methoxyphenyl hydrazone (FCCP), and the inhibitors rotenone plus antimycin A (Rot/AA) were tested according to manufacturer’s manual. For subsequent Mito Stress Test, optimal cell numbers and FCCP concentrations were used based on the previously carried out cell characterization. The appropriate cell numbers (SCL-1: 15,000, NHEK: 20,000) were seeded in a Seahorse 96-well plate and the sensor was equilibrated in Seahorse equilibration buffer in a CO_2_-free incubator overnight. All cells were washed and mock treated or treated with different concentrations of GP in Seahorse medium (eight replicates/conditions) and were incubated for 1 h in a CO_2_-free incubator at 37 °C. Meanwhile, the cartridge was loaded with appropriate concentrations of Oligo [Port A; SCL-1: 2 µM (final conc.), NHEK: 2 µM (final conc.)], FCCP [Port B; SCL-1: 0.5 µM (final conc.), NHEK: 2 µM (final conc.)], and Rot/AA [Port C; SCL-1: 0.5 µM (final conc.), NHEK: 0.5 µM (final conc.)]. After equilibration of the sensor, the assay was performed as described above. For data analysis, the Agilent Seahorse Wave Desktop software was used. The raw data were normalized to the corresponding Hoechst staining. The program calculated the parameters based on the OCR, including basal, maximal and non-mitochondrial respiration, SRC, proton leak, and ATP production. The percentage of each parameter was referred to mock-treated cells.

### Caspase activity assay

Caspase activity assays were performed according to the manufacturer’s specification. In principle, a selective substrate for the used caspase was added to the cells, which can be cleaved by active caspases. The formation of the fluorescent product was measured and an increase in fluorescence correlated with the activation of caspases upon stimulation of apoptosis. Caspase 8 activity representing the extrinsic pathway and caspase 9 activity representing the intrinsic pathway as well as the activity of the executioner caspases 3/7 were analyzed. Cells were grown to subconfluence in 96-well plates and incubated with different concentrations of GP or mock treated for 6 and 24 h, respectively. Staurosporine (Sts) at a concentration of 20 µM served as positive control. The substrate caspase working solution (3/7, 8, or 9) was prepared and added for 1 h. The inhibitor zVAD-(OMe)-FMK (zVAD) was added 10 min prior to the end of the incubation time to prevent further cleavage of the substrate. Cell-free wells served as background control. Subsequently, the fluorescence was measured using a microplate reader under different emission (Em) and excitations (Ex) wavelengths dependent on the caspase (caspase 3/7: Ex 360 nm, Em 470 nm; caspase 8: Ex 370 nm, Em 450 nm; caspase 9: Ex 375, Em 435 nm). For quantification, the background values were subtracted, the mock-treated control was set at 1.0, and the GP treatment was calculated in relation to the control.

### SDS-PAGE and western blot analysis

For sodium dodecyl sulfate polyacrylamide gel electrophoresis (SDS) and western blotting (Laemmli 1970), GP and mock-treated cells were lysed in 1% SDS (Roth, Karlsruhe, Germany) with 0.1% diluted protease inhibitor cocktail and sonicated. Protein concentration was determined using the DC™ Protein Assay Kit. For each sample, 20 μg protein was mixed with 4xSDS-PAGE sample buffer (40% glycerol, 20% β-mercaptoethanol, 12% SDS, 0.4% bromophenol blue) and heated at 95 °C for 10 min. Subsequently, the samples were subjected to 12% or 15% (w/v), SDS-polyacrylamide gels, respectively. After blotting the proteins onto polyvinylidene difluoride (PVDF) membranes (GE Healthcare, Solingen, Germany) and blocking (5% (w/v) milk powder), incubation with the primary (1:1000) and secondary antibody (1:15,000) was performed, the blot developed using the ECL-system (Cell Signaling Technology), and monitored by the Fusion SL Advance gel documentation device (Peqlab, Erlangen, Germany). Quantification of proteins was done using the FusionCapt Advance software.

### Immunostaining

Immunofluorescence experiments were performed to visualize the protein of interest. Cells were grown until a confluence of 50–70% in six-well plates containing coverslips and, then, mock treated or treated with GP. After incubation and washing with PBS, cells were fixed with preheated (37 °C) 4% paraformaldehyde (PFA) for 20 min at RT. After washing with PBS, 1 ml Triton-X-100 (0.15% in PBS) was added for 15 min to permeabilize the cells. Subsequently, the cells were blocked in 10% normal goat serum (NGS) in PBS for further 15 min. The adequate primary antibody was diluted 1:100 in 1% NGS in PBS and 100 µl added to each sample. After incubation at 4 °C overnight and washing with PBS, the secondary antibody (100 µl/sample) at a concentration of 0.1% in 1% NGS in PBS was added and incubated for 1 h in the dark. After discarding the antibody solution and three washing steps with PBS for 10 min in the dark, the coverslips were fixed on microscopic slides using ProLong™ Gold Antifade Reagent with DAPI. The immunofluorescence of pRIP3 and RIP3 after GP treatment was recorded using an Ultraview spinning disc confocal microscope (PerkinElmer Corporation). The intensity of the signals was calculated using Image J software (Wayne Rasband, NIH). At least ten pictures of each condition were quantified. The ratio of pRIP3 to RIP3 was determined and mock-treated control was set at 1.0.

### Trypan blue staining

For determination of the cell membrane integrity, the trypan blue exclusion method was performed (Tran et al. 2011). Here, cells were grown in six-well plates to subconfluence. The cells were mock treated or treated with different concentrations of GP alone or in combination with necrostatin-1 (Nec1), an inhibitor of necroptosis. The detergent Triton-X-100 (500 ppm) was used as positive control. After incubation, cells were washed with PBS and incubated with 200 µl of trypsin for 5 min in a humidified atmosphere (37 °C, 5% CO_2_) to detach the cells, which were collected in an Eppendorf tube. The reaction was stopped by adding 800 µl growth medium. After mixing, 10 µl of the cell suspension was mixed with 10 µl of a 0.4% trypan blue solution and 10 µl of this was used to determine the number of unstained and blue colored cells by means of a Neubauer counting chamber. For evaluation, the total cell number was calculated and the percentage of cells with an intact and permeabilized cell membrane was determined.

### Extracellular LDH measurement

Rupture of the cell membrane is a marker of necrotic and necroptotic cell death (Zhang et al. 2018). To measure this, the occurrence of extracellular amounts of the cytosolic lactate dehydrogenase can be measured using a LDH ELISA kit. Subconfluent cells were mock treated or treated with GP for 6, 24, and 48 h. After incubation, the supernatant was collected and centrifuged at 2000×g for 10 min at RT. The supernatant (50 µl/well) was used for the LDH ELISA. The assay was performed in accordance with the manufacturer’s protocol. For quantification, the cell number was determined using a Neubauer counting chamber. A standard curve was included in each experiment. The absorbance was measured at 450 nm and the amount of extracellular LDH was derived from the standard curve. The mock-treated control was set at 1.0 and all other conditions were normalized to it.

### Statistics

Means were calculated from at least three independent experiments (*n* ≥ 3), unless otherwise stated. Error bars represent the standard error of mean (SEM). Statistical analysis was performed by one-way ANOVA with post hoc test (Dunnett or Bonferroni) or student’s *t* test using GraphPad Prism 5 software; *p ≤ 0.05, **p ≤ 0.01 and ***p ≤ 0.001 were chosen as levels of significance.

## Results

### High expression of anti-apoptotic BCL-2 proteins in tumor cells

**Fig. 1:**
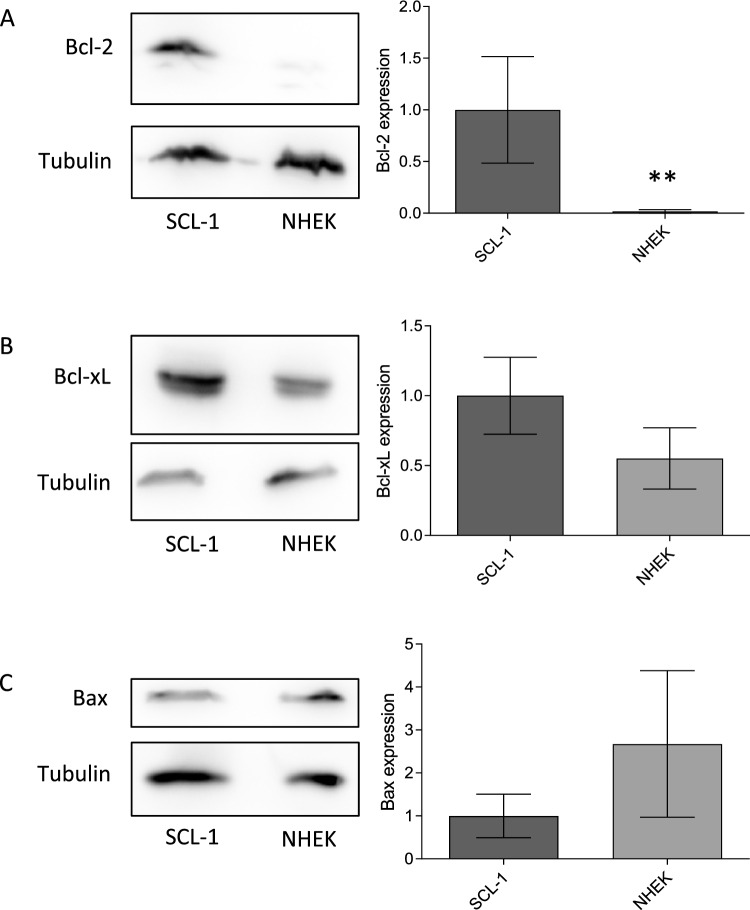
Expression of anti-apoptotic and pro-apoptotic proteins. Basal protein expression of anti-apoptotic (BCL-2, BCl-xL) and pro-apoptotic (BAX) proteins in SCL-1 carcinoma cells and normal keratinocytes was determined by western blot. Representative blots were depicted. Tubulin served as loading control. **A–C** Densitometric analysis of the western blots was performed. The protein amount was set in relation to the respective loading control and the protein level of SCL-1 cells was set at 1.0. Data represent means ± SEM of three independent experiment (*n* = 3). Student’s *t* test was used for the determination of statistical significance; **p < 0.01

An evasion of cell death is described for several tumor types due to the disruption of the intrinsic pathway of apoptosis and/or an imbalance of the expression of pro- and anti-apoptotic proteins toward an increase in the amount of anti-apoptotic BCL-2 proteins (Adams and Cory 2007; Hanahan and Weinberg 2011; Sharma et al. 2019). To find out whether the skin tumor cell line SCL-1 exhibits a higher amount of anti-apoptotic proteins, the basal amount of BCL-2 and BCL-xL as well as the amount of the pro-apoptotic protein BAX was determined by western blots and compared with normal human epidermal keratinocytes (NHEK) (Fig. 1). The densitometric analysis of three independent experiments resulted in a significant increase in the amount of BCL-2 in SCL-1 cells compared with NHEK (Fig. 1A). Furthermore, the BCL-xL protein level was also moderately elevated in the tumor cells compared with NHEK (Fig. 1B). The expression of BAX was virtually similar in SCL-1 cells and normal keratinocytes (Fig. 1C).

### ABT-199 lowered cell viability in both cell types while GP showed selective toxicity

**Fig. 2:**
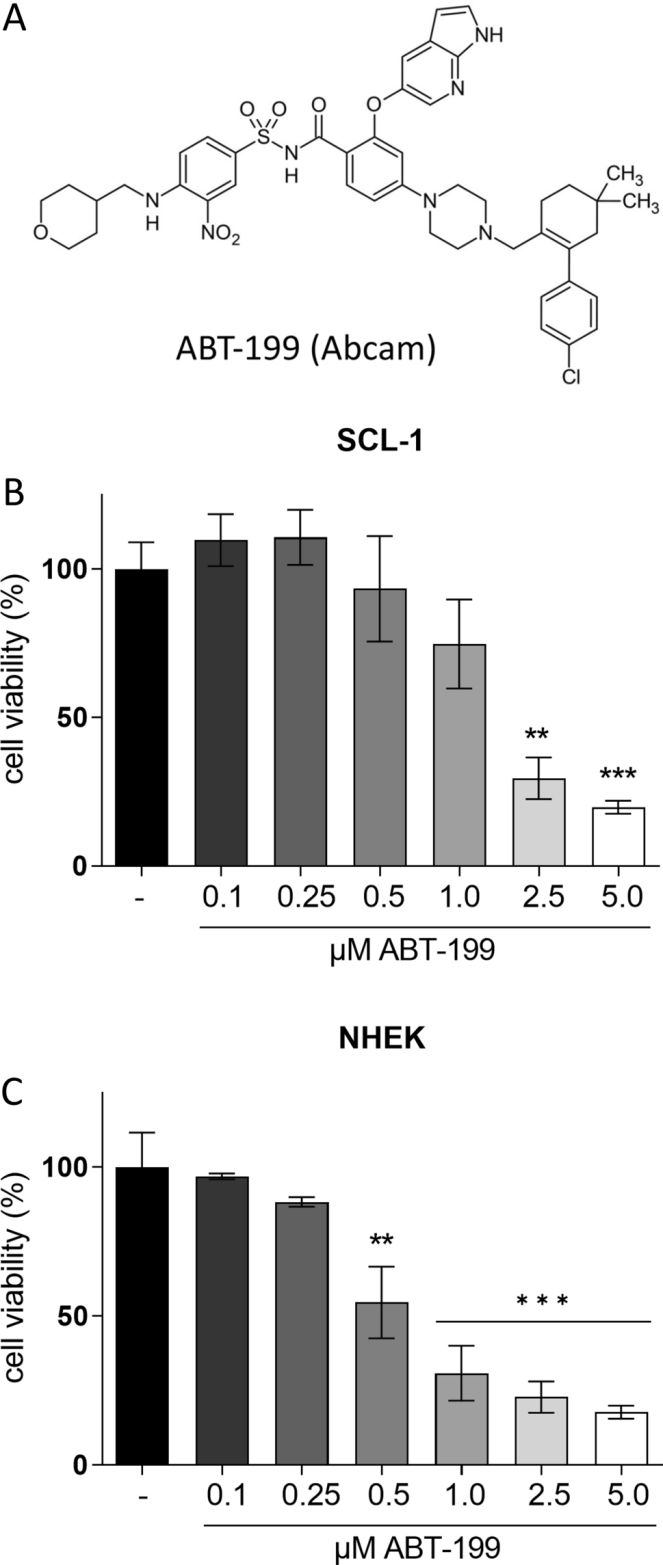
Effect of ABT-199 on cell viability of SCL-1 and NHEK. **A** Chemical structure of ABT-199/venetoclax. To determine the effect of ABT-199 on cell viability, SCL-1 carcinoma cells **(B)** and keratinocytes (NHEK) **(C)** were treated with different concentrations of ABT-199 for 96 h. Cell viability was measured by MTT assay. Mock-treated control was set at 100%. Data represent means ± SEM of at least three independent experiments (*n* ≥ 3). One-way ANOVA with Dunnett’s multiple comparison test was used for the determination of statistical significance compared to mock-treated control; **p < 0.01, ***p < 0.001

As epidemiological studies also showed that the expression of anti-apoptotic BCL-2 can be higher than the expression of BCL-xL and MCL-1 in skin cancer cells (D’Aguanno and del Bufalo 2020), BH3 mimetic substances are in the focus of research and clinical trials (Kehr and Vogler 2021). ABT-199/venetoclax (Fig. 2A) is a selective and the first FDA-approved BH3 mimetic binding to the BH3 binding groove of BCL-2 with high affinity and being described to be active in numerous cancer types (Vaillant et al. 2013; Bose et al. 2017; Hafezi and Rahmani 2021). The effect of ABT-199 on cell viability was determined in both tumor and normal cells. For that, the cells were treated with varying concentrations of the compound for 96 h and cell viability was measured. ABT-199 decreased cell viability in a dose-dependent manner in SCL-1 carcinoma cells (Fig. 2B) and NHEK (Fig. 2C). However, ABT-199 showed a similar effect on normal as well as on tumor cells, which is reflected in the calculated IC_50_ values (via non-linear curve fit analysis) of 1.8 µM for SCL-1 and of 0.7 µM for the normal keratinocytes. In context of a minimization of toxic effects on normal cells being responsible for numerous side effects in patients, ABT-199 was not used for further in vitro experiments as normal keratinocytes responded even more sensitive to this compound than the tumor cells (Fig. 2C).

**Fig. 3:**
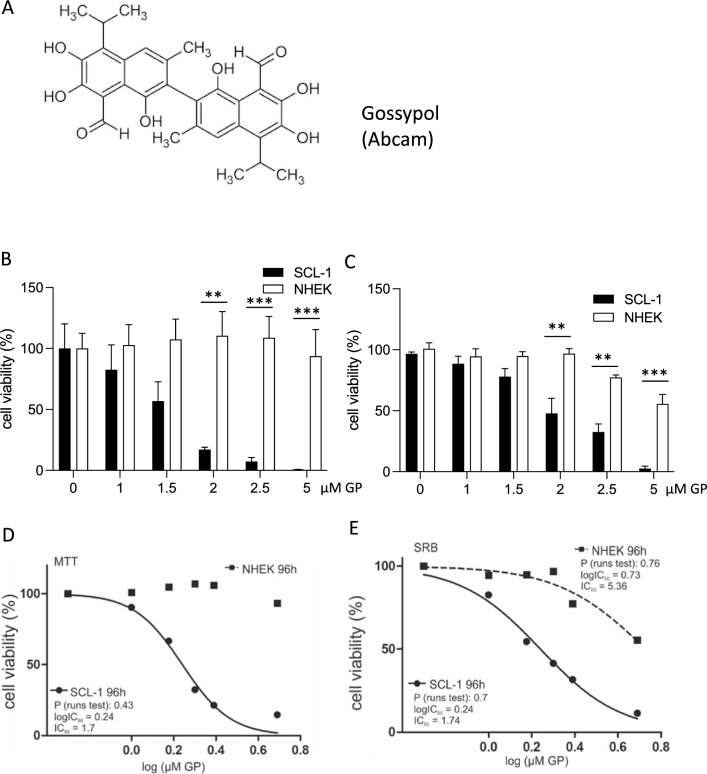
Selective effect of GP on cell viability of skin cells. **A** Chemical structure of gossypol (GP). To examine the effect of GP on cell viability, SCL-1 carcinoma cells and keratinocytes (NHEK) were treated with different concentrations of GP for 96 h or mock treated (0) and cell viability was measured by MTT **(B)** and SRB **(C)** assay. Mock-treated controls were set at 100%. Data represent means ± SEM of at least three independent experiments (*n* ≥ 3). Student’s *t* test was used for the determination of statistical significance; **p < 0.01, ***p < 0.001. **D, E** IC_50_ values were calculated by non-linear curve fit analysis (GraphPad Prism 5) based on MTT **(D)** and SRB assay **(E)**

The natural compound gossypol (GP, Fig. 3A) consists of (+) and (−) enantiomers and acts as pan-Bcl-2 inhibitor (Liu et al. 2022). We could show earlier (Haasler et al. 2021), that GP exerted a selective toxicity on melanoma cells, but had no toxic effect on normal (healthy) cells such as melanocytes. Therefore, the effect of GP on cell viability of squamous SCL-1 and normal keratinocytes was tested. After 96 h, cell viability of SCL-1 carcinoma cells and NHEK was measured by MTT (Fig. 3B) and SRB assay (Fig. 3C). GP lowered cell viability of the tumor cell line in a dose-dependent manner compared to the mock-treated controls. A significant loss of cell viability was determined in the higher concentration range between 2 and 5 µM GP. In contrast to that, only the highest concentration of 5 µM lowered cell viability of NHEK (Fig. 3C, SRB assay). Nevertheless, the viability of NHEK was still significantly higher than for SCL-1 cells. The calculated SCL-1 IC_50_ values of MTT (Fig. 3D) and SRB data (Fig. 3E) were almost identical, 1.7 µM versus 1.74 µM. As GP exerted a selective toxicity on the studied cell types, the pan-BCL-2 inhibitor GP was used for further studies at a concentration of 2.5 µM in most cases, and in some experimental approaches, a concentration of 5 µM GP was included as well.

### Similar uptake of GP in tumor and normal cells

**Fig. 4:**
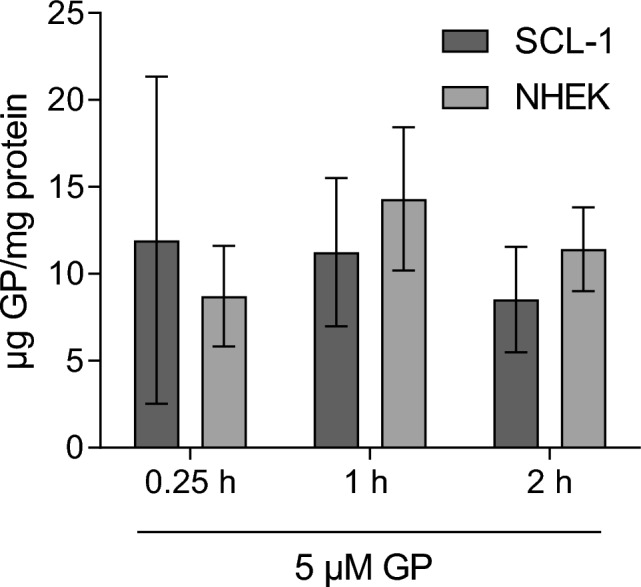
Intracellular amount of GP in SCL-1 cells and keratinocytes (NHEK). Cells were harvested after treatment with 5 µM GP for 0.25, 1, and 2 h and analyzed by HPLC. The amount of intracellular GP was related to the respective protein level. Experiments were performed in triplicate (*n* = 3)

The experiments regarding cytotoxicity of GP showed a selective effect of this compound. To examine whether that effect is due to differences in uptake, the concentration of GP within the cells was determined by HPLC. Therefore, SCL-1 carcinoma cells and NHEK were incubated with 5 µM GP for 0.25, 1, and 2 h. The results are shown in Fig. 4. The average of intracellular concentrations of GP was about 10–12 µg GP per mg protein. The detectable amount of GP (t = 0.25 h) within the cells related to the amount of GP applied was on the average of 15%. There was no difference in the absorbed GP amount between tumor and normal cells, which was identical to the earlier published data on melanoma cells and melanocytes (Haasler et al. 2021).

### Decreased mitochondrial membrane potential and increased fragmentation by GP

**Fig. 5:**
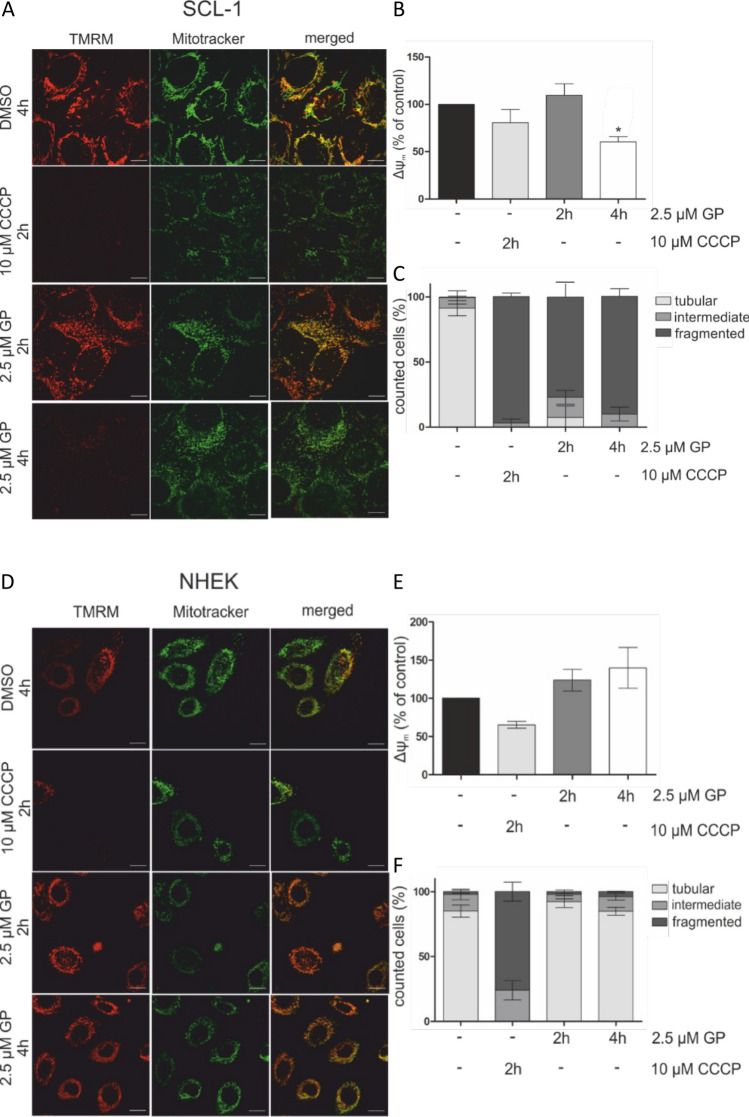
Effect of GP on mitochondrial membrane potential (∆ψ_m_) and mitochondrial fragmentation in SCL-1 cells and NHEK. Subconfluent SCL-1 cells **(A)** and NHEK **(B)** were mock treated (−) or treated with 2.5 µM GP for 2 or 4 h. CCCP at a concentration of 10 µM (2 h) served as positive control. Subsequently, cells were incubated with 100 nM TMRM and 100 nM MitoTRACKER™ Green for 30 min and analyzed by confocal microscopy. The scale bar is 20 µm. **A, D** Representative pictures are shown. **B, E** Calculation of the intensity of TMRM to MitoTRACKER™ Green was performed using Image J. The mock-treated control was set at 100%. One-way ANOVA with Dunnett’s multiple comparison test was used for the determination of statistical significance; *p < 0.05. **C, F** For quantification of mitochondrial morphology/fragmentation (Duvezin-Caubet et al. 2006), at least 30 cells of each condition were counted. Data represent means ± SEM of three independent experiments (*n* = 3)

Changes in mitochondrial membrane potential (∆ψ_m_) and/or mitochondrial membrane permeability (MMP) often correlate with mitochondrial dysfunction (e.g., fragmentation), changes in oxidative phosphorylation (OXPHOS), and activation of cell death mechanisms (Landes and Martinou 2011; Bock and Tait 2020). As BH3 mimetic substances have been shown to affect the mitochondrial membrane potential (∆ψ_m_) (Henz et al. 2019), a GP-mediated modulation of ∆ψ_m_ and mitochondrial dysfunction was investigated (Fig. 5). GP significantly diminished ∆ψ_m_ in SCL-1 cells after a 4 h treatment at a concentration of 2.5 µM GP (Fig. 5A, B) compared to mock-treated cells. In contrast, no decrease of ∆ψ_m_ was observed in NHEK (Fig. 5D, E). In tendency, ∆ψ_m_ slightly increased in the normal (healthy) cells after 4 h. With regard to a reported correlation between a drop in ∆ψ_m_ and a change in mitochondrial fragmentation being a measure for mitochondrial dysfunction (Tang et al. 2018), the mitochondrial morphology was assessed after GP treatment of SCL-1 and NHEK. GP induced a fragmentation in about 80% of SCL-1 cells (Fig. 5C) after 2 h which was further increased over 95% after 4 h. This finding was similar to the OXPHOS uncoupler carbonyl cyanide m-chlorophenyl hydrazone (CCCP)-treated cells, which served as positive control. As opposed to the tumor cells, no change in mitochondrial morphology after GP treatment was determined in NHEK (Fig. 5F) compared to the mock-treated cells. On the contrary, CCCP treatment also resulted in an increased mitochondrial fragmentation in NHEK.

### GP decreases OXPHOS in squamous skin cancer cells

**Fig. 6:**
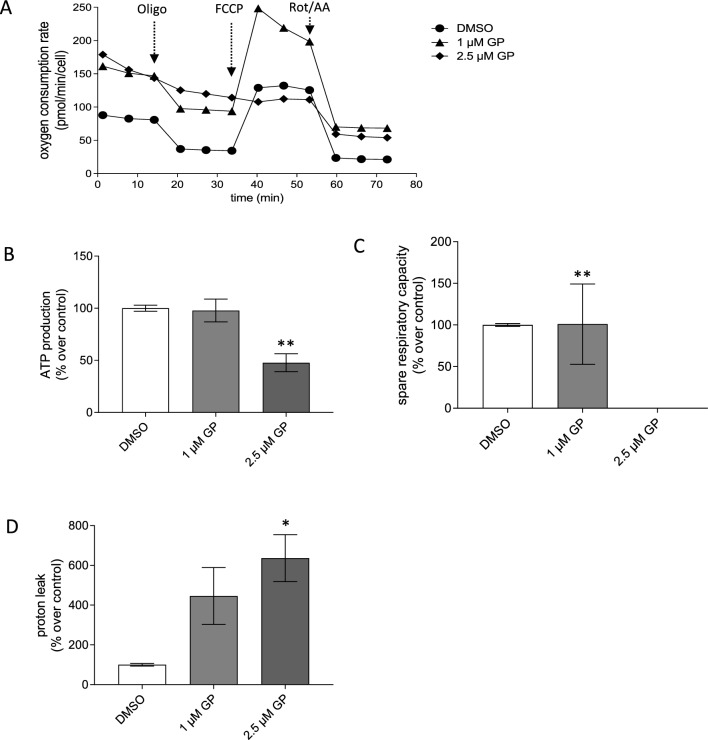
Mitochondrial respiration of GP-treated SCL-1 carcinoma cells. **A** After treatment with different concentrations of GP for 1 h or mock treated (DMSO), the oxygen consumption rate (OCR) was measured after successive injection of oligomycin (Oligo), FCCP, and rotenone/antiymcin A (Rot/AA) by Seahorse XF Analyzer. Representative curves were depicted. **B–D** Based on the OCR in response to these mitochondrial stressors, ATP production **(B)**, spare respiratory capacity **(C),** and proton leak **(D)** were calculated. The values of mock treated cells were set at 100%. Data represent means ± SEM of three independent experiments (*n* = 3). One-way ANOVA with Dunnett’s multiple comparison test was used for the determination of statistical significance between mock treated and GP-treated cells; *p < 0.05, and **p < 0.01

**Fig. 7:**
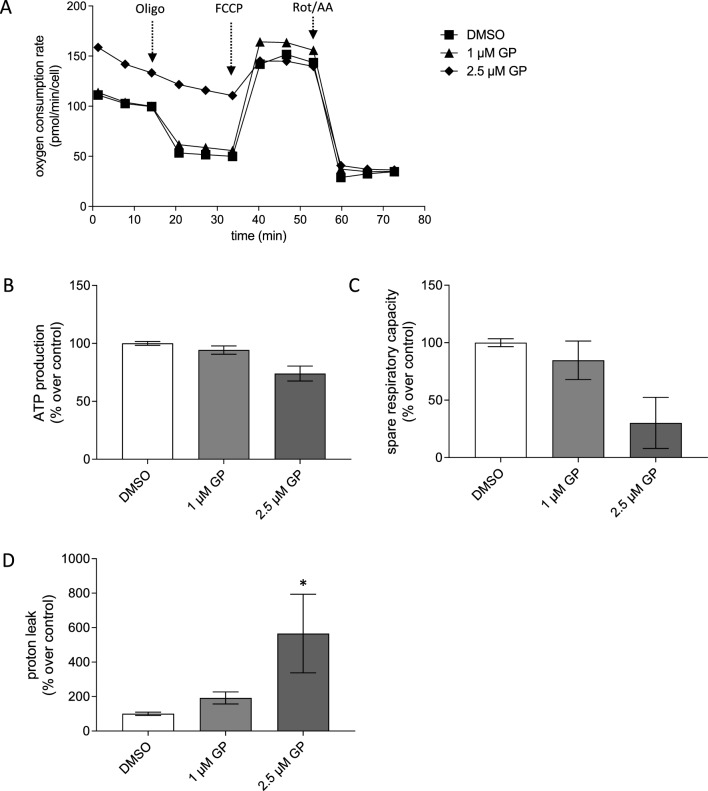
Mitochondrial respiration of GP-treated normal keratinocytes (NHEK).** A** After treatment with different concentrations of GP or mock treated for 1 h, the oxygen consumption rate (OCR) was measured after successive injection of oligomycin (Oligo), FCCP, and rotenone/antiymcin A (Rot/AA) by Seahorse XF Analyzer. Representative curves are depicted. **B–D** Based on the OCR in response to these mitochondrial stressors, ATP production **(B)**, spare respiratory capacity **(C),** and proton leak **(D)** were calculated. The values of untreated cells were set at 100%. Data represent means ± SEM of three independent experiments (*n* = 3). One-way ANOVA with Dunnett’s multiple comparison test was used for the determination of statistical significance between mock treated and GP-treated cells; *p < 0.01

As the energy production by OXPHOS requires an intact ∆ψ_m_ (Zorova et al. 2018), the effect of GP on mitochondrial respiration was determined using Seahorse XF Analyzer. As the drop in ∆ψ_m_ occurred rapidly after GP was added to the SCL-1 cells and the IC_50_ value of the GP-treated SCL-1 cells was calculated to be 2.8 µM at 24 h post-treatment, concentrations < 2.8 µM GP for oxygen consumption rate (OCR) measurements were used. The tumor cells and normal keratinocytes were pre-incubated for 1 h with 1.0 and 2.5 µM GP before the Mito Stress Tests were performed (Gu et al. 2021). The assay provides information about oxygen consumption rate (OCR) after the addition of different mitochondrial stressors including oligomycin (Oligo), the uncoupler FCCP, and rotenone/antimycin A (Rot/AA), from which the bioenergetic parameters (Figs. 6, 7) can be calculated such as basal respiration, ATP production, spare respiratory capacity (SRC; a measure of the cell’s ability to respond to energetic demand), and proton leak, meaning that some protons “leak” back across the mitochondrial inner membrane due to damaged mitochondria and/or to maintain the activity of the respiratory chain (Divakaruni et al. 2014; Darcy et al. 2020; Marchetti et al. 2020).

The basal respiration rather tended to increase to 1.7-fold and twofold in SCL-1 carcinoma cells after application of 1.0 and 2.5 µM GP compared to mock treated controls (Fig. 6A). At a concentration of 2.5 µM GP, the ATP production was lowered to 50% compared to control level (Fig. 6B), the spare respiratory capacity was completely lost (Fig. 6C), and the proton leak significantly increased (Fig. 6D). Unlike the effect of GP on respiration in tumor cells, only minor effects were observed in normal (healthy) keratinocytes. Even though the highest GP concentration of 2.5 µM also increased the basal respiration of NHEK (Fig. 7A), the ATP production was unaffected after GP treatment at the studied concentrations (Fig. 7B). In addition, the SRC was lowered after treatment with 2.5 µM GP, but this effect was not significant compared to mock treated cells (Fig. 7C). At the highest concentration of 2.5 µM, the proton leak significantly raised similar to the rate in the squamous tumor cells. Interestingly, the proton leak of NHEK was about half of the value of the SCL-1 cells after treatment with 1 µM GP (Fig. 7D). In summary, treatment of the SCL-1 carcinoma cells with GP resulted in a decrease of both ATP production and SRC and an increase in the proton leak.

### GP does not initiate apoptosis in SCL-1 tumor cells

**Fig. 8:**
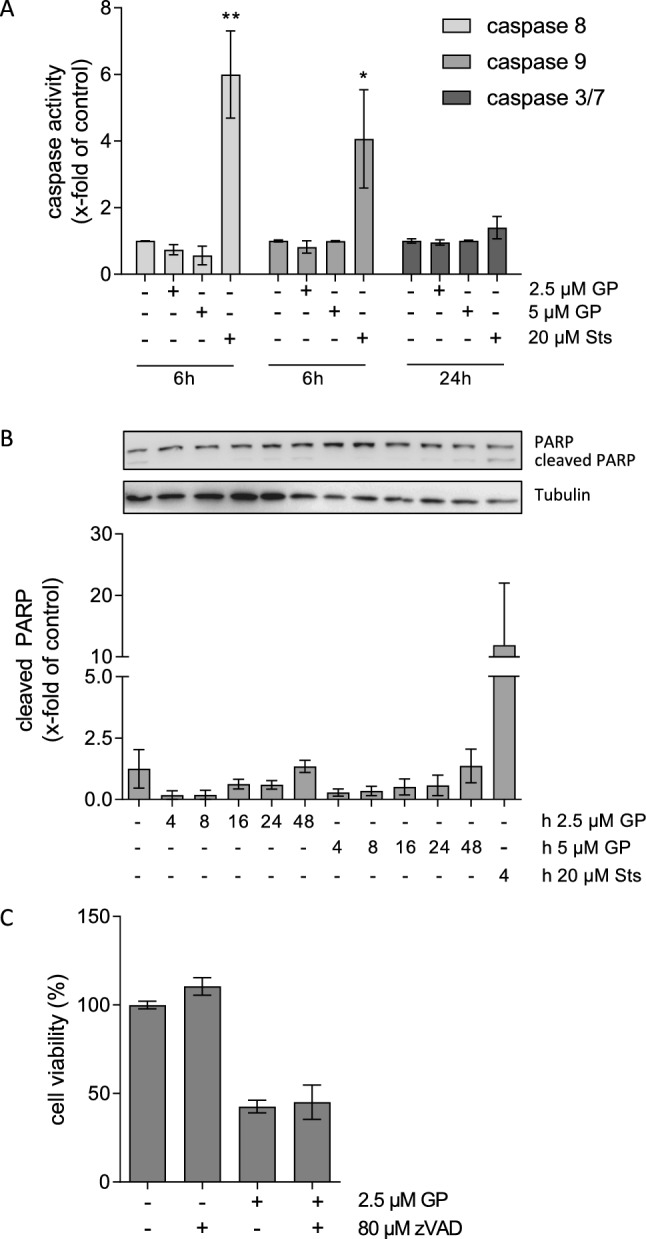
Effect of GP on apoptosis in SCL-1 carcinoma cells. **A** After treatment with 2.5 or 5 µM GP or mock treated (-) for 6 or 24 h, caspase activity was measured. Staurosporine (Sts, 20 µM) served as positive control. Mock treated control was set at 1.0. Data represent means ± SEM of three independent experiments (*n* = 3). One-way ANOVA with Dunnett’s multiple comparison test was used for the determination of statistical significance; *p < 0.05, **p < 0.01. **B** Subconfluent cells were treated with 2.5 or 5 µM GP for 4, 8, 16, 24, and 48 h. Mock treated cells were used as negative control, 20 µM staurosporine (Sts) served as positive control. Protein expression of full length and cleaved PARP was determined by western blot. Beta-tubulin was used as loading control. A representative blot of three independent experiments was depicted (*n* = 3). Quantification of the protein amount was calculated compared to the mock-treated control. Mock treated control was set at 1.0. **C** For rescue experiment, subconfluent cells were pre-treated for 4 h with 80 µM of the pan-caspase inhibitor zVAD, followed by GP (2.5 µM) treatment for further 24 h. Cell viability was measured by MTT assay. Mock treated control was set at 100%. Data represent means ± SEM of three independent experiments (*n* = 3)

As described above, changes in mitochondrial membrane potential may result in apoptotic cell death. In earlier studies, we could show that GP resulted in apoptotic cell death of A375 melanoma cells (Haasler et al. 2021). In that context and due to the potential of GP inducing a cytotoxic effect on SCL-1 carcinoma cells and not on NHEK, apoptosis markers such as the activity of the initiator caspases 8 and 9, the executioner caspases 3 and 7, and poly (ADP-ribose) polymerase (PARP) cleavage were studied after treating the tumor cells with 2.5 and 5 µM GP or 20 µM staurosporine (Sts) as positive control (Fig. 8). The activity of the initiator caspases was measured after 6 h and the activity of the executioner caspases 24 h after treatment, based on earlier published data with melanoma cells (Haasler et al. 2021). In contrast to melanoma cells, GP neither activated the initiator caspases 8 or 9 nor the executioner caspases 3 and 7. Conversely, Sts significantly increased the activity of caspase 8 and 9, but only a slight increase of caspase 3/7 activity was measured (Fig. 8A). To verify these data, the expression level of the apoptosis relevant protein PARP was determined by western blot analysis. For this purpose, SCL-1 carcinoma cells were treated with 2.5 and 5 µM GP up to 48 h to measure the protein level of full length and cleaved PARP. At 48 h post-treatment, the time course analysis indicated a significant loss of cell viability (≤ 50%) at the used concentrations (data not shown). In contrast to the Sts control, the cleaved PARP level in GP-treated cells did not exceed the protein level of the mock-treated cells (Fig. 8B). To finally demonstrate a GP-initiated caspase and PARP-independent cell death of SCL-1 carcinoma cells, the cells were incubated with GP combined with the pan-caspase inhibitor zVAD(OMe)-FMK. In contrast to melanoma cells (Haasler et al. 2021), the induced cytotoxic effect of GP on SCL-1 tumor cells did not change in the presence of zVAD indicating that the cell death occurred independent of apoptosis (Fig. 8C).

### GP initiates necroptosis in SCL-1 tumor cells

**Fig. 9:**
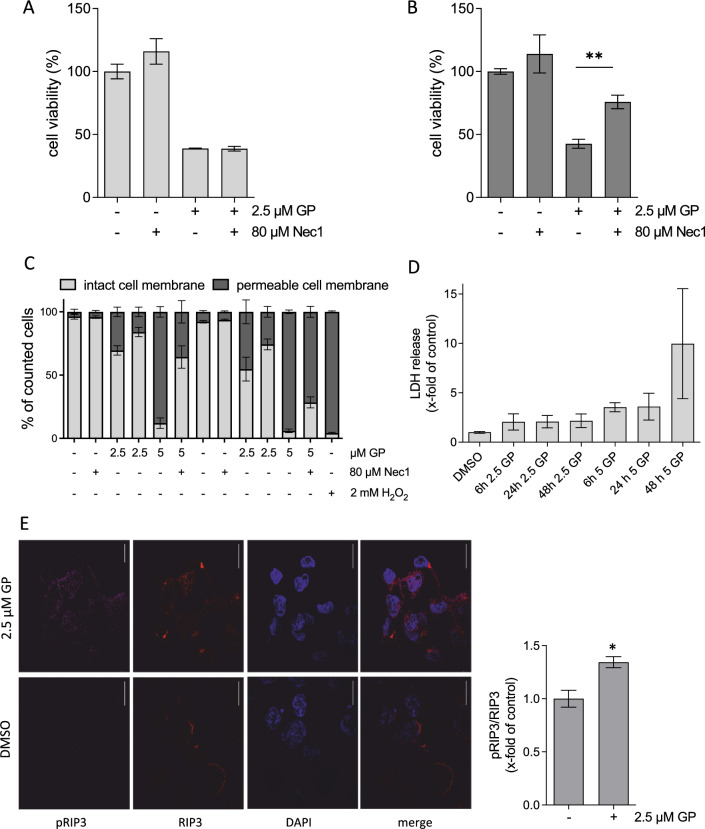
Effect of GP on necroptosis in SCL-1 carcinoma cells. **A, B** After pre-incubation with 80 µM of the necroptosis inhibitor necrostatin-1 (Nec1) for 4 h, cells were mock treated (-) or 2.5 µM GP was added to A375 melanoma (**A**) and SCL-1 carcinoma cells (**B**) for further 24 h. Cell viability was measured by MTT assay. Mock treated cells were set at 100%. Data represent means ± SEM of three independent experiments (*n* = 3). Student’s *t* test was used for determination of statistical significance; **p < 0.01. Subconfluent cells were mock treated (−) or pre-treated with 80 µM Nec1 for 4 h, followed by treatment with 2.5 and 5 µM GP for further 24 and 48 h. H_2_O_2_ (2 mM) was used as positive control. Subsequently, the number of cells was counted using trypan blue staining. Blue colored cells were defined as having a permeable membrane. The total cell number of each condition was set at 100% and the percentage of cells with an intact versus a permeable membrane is shown. **D** After treatment of subconfluent cells with 2.5 and 5 µM GP for 6, 24, and 48 h, the LDH content in the supernatant was measured. Data represent x-fold increase of extracellular LDH compared to mock treated control (DMSO), which was set at 1.0. One-way ANOVA with Dunnett’s multiple comparison test was used for the determination of statistical significance (*n* = 4). **E** After incubation without (DMSO) and with 2.5 µM GP for 24 h, immunostaining against phosphorylated RIP3 (pRIP3) and unphosphorylated RIP3 (RIP3) was performed. DAPI was used for nucleus staining. Representative pictures are shown. Scale bars represent 20 µm. For quantification, the fluorescence intensity of pRIP3 was set in relation to RIP3. Mock treated control (-, represents DMSO) was set at 1. Data represent means ± SEM of three independent experiments (*n* = 3). Student’s *t* test was used for the determination of statistical significance; *p < 0.05

None of the above tested apoptotic markers could be detected in SCL-1 cells. There is increasing evidence that necroptosis is a back-up cell death pathway to kill tumor cells efficiently (Chen et al. 2016). The molecular mechanisms are based on a signal cascade mediated by receptor-interacting serine/threonine protein kinase (RIP) 1 and RIP3, finally resulting in an increase in membrane permeability and release of lactate dehydrogenase (Gong et al. 2019; Kim et al. 2021). To figure out whether the necroptotic pathway was activated after GP treatment in SCL-1 carcinoma cells, necrostatin-1 (Nec1), a cell-permeable and selective inhibitor of the RIP1 kinase and downstream phosphorylation of RIP3 was tested, which finally interrupts the mechanisms required for necroptotic cell death (Cao and Mu 2021). SCL-1 carcinoma cells were pre-treated with Nec1 prior to the GP treatment. The GP-induced decrease in cell viability was significantly diminished in the presence of Nec1, indicating that necroptosis could be involved (Fig. 9B). However, no complete recovery was seen with Nec-1, suggesting that GP might stimulate additional mechanisms resulting in cell death. As proof of principle, Nec1 was also tested in A375 melanoma cells. Nec1 had no impact on A375 cell viability (Fig. 9A), which can be killed by GP-mediated apoptosis (Haasler et al. 2021). To determine whether GP has an impact on the permeabilization of the cellular membrane, trypan blue staining was performed in SCL-1 carcinoma cells. The treatment with Triton-X-100 resulted in 100% trypan blue positive cells (data not shown). At a concentration of 2.5 µM GP, around 30% of the counted cells had permeabilized cell membranes after 24 h, which further increased up to 50% after 48 h. The higher doses of 5 µM GP enhanced this effect resulting in nearly 90% of tumor cells with permeabilized cell membranes after 48 h (Fig. 9C). To demonstrate that this effect was due to necroptosis, a combination treatment with Nec1 was performed. Nec1 diminished the extent of damaged cells by GP after 24 and 48 h indicating that necroptosis was involved in the cytotoxic effect of GP on SCL-1 carcinoma cells (Fig. 9C). As a consequence of the permeabilized membranes, cellular contents, such as lactate dehydrogenase (LDH), can be released. Therefore, the extracellular amount of LDH was measured after GP treatment for 6, 24, and 48 h in SCL-1 carcinoma cells. In fact, the LDH amount increased after the treatment of 2.5 µM GP at the studied time points, which was further enhanced using 5 µM GP (Fig. 9D). As both the increase in membrane permeability and LDH release are late markers of necroptosis, an early key event had to be examined as well. In this context, RIP kinases play a crucial role. After dissociation of RIP1 from a complex including inactive caspase 8, the protein recruits RIP3 followed by phosphorylation of RIP3 (Gong et al. 2019). For this reason, the phosphorylation of RIP3 was determined in GP-treated SCL-1 carcinoma cells. GP at a concentration of 2.5 µM significantly elevated the phosphorylation of RIP3 in relation to unphosphorylated RIP3 after 24 h (Fig. 9E). Moreover, the localization of unphosphorylated RIP3 changed after GP treatment. Under basal conditions, RIP3 was predominantly located around the nucleus, whereas it was uniformly distributed in the presence of GP (Fig. 9E). In summary, the data indicate that treatment of SCL-1 carcinoma cells with GP resulted at least in part in a necroptotic cell death.

## Discussion

In context of cutaneous squamous cell carcinoma, a systemic and/or targeted therapy is the treatment of choice in the majority of cases of advanced unresectable or metastatic cSCC (Ribero et al. 2017; Corchado-Cobos et al. 2020). However, the response rate is rather modest in context of resistance, recurrence, and severe side effects (Marti et al. 2016; Agirgol et al. 2020). An interesting alternative is the use of BH3 mimetics to antagonize overexpressed anti-apoptotic BCL-2 proteins to release pro-apoptotic proteins (Vogler 2014; Townsend et al. 2021). In our study, BCL-xL was overexpressed in SCL-1 cells compared to normal (healthy) cells, which is in line with data of patients with cutaneous squamous cell carcinoma (cSCC) having elevated BCL-xL levels as well (Vasiljević et al. 2009). Numerous validated and putative BH3 mimetics are described to be in use for in vitro studies and clinical trials, but most of them deal with melanoma and head and neck squamous cell carcinoma (Swiecicki et al. 2016; Melo et al. 2022). Currently, very little is known about the effect of BCL-2 inhibitors on squamous cell carcinoma of the skin. Only one publication reported that cSCC cells are more sensitive to the BCL-2 inhibitor ABT-737, if the BCL-2 family member MCL-1 was suppressed in these cells (Geserick et al. 2015). For our studies on squamous skin carcinoma, the FDA-approved drug ABT-199/venetoclax and the natural compound (±) gossypol (GP) have been tested. ABT-199 was described to be effective in the treatment of specific types of leukemia (Cang et al. 2015; Thol and Ganser 2020). However, side effects such as skin rash, vitiligo, cytopenia, and tumor lysis syndrome were described as well (Abdeen et al. 2022). Our in vitro data showed that ABT-199 also has a toxic effect on normal skin cells in contrast to GP showing a selective toxicity on the studied squamous skin carcinoma cells. These data are in line with earlier published data on the effect of GP on melanoma cells (Haasler et al. 2021).

Decades ago, it was already described that gossypol has a modulatory effect on the mitochondrial membrane potential (Martínez 1992; Barhoumi 1996). This effect seemed to fall into oblivion for some time, even though changes in the mitochondrial membrane potential or permeability affect mitochondrial dynamics, result in mitochondrial dysfunction, and trigger cell death pathways (Landes and Martinou 2011; Bock and Tait 2020). As BH3 mimetics, AT-101 and GP initiated mitochondrial dysfunction and different types of cell death such as mitophagic or autophagic cell death as well as apoptosis in glioma and colon cancer cells (Lu et al. 2017; Meyer et al. 2018), GP was tested on modulation of the mitochondrial membrane potential (∆ψ_m_) of the cSCC cell line SCL-1 and normal keratinocytes. ∆ψ_m_ was significantly lowered and the number of fragmented mitochondria increased by GP in the tumor cells in contrast to the normal cells. In this regard, ABT-199, S63845 (MCL-1 inhibitor), and A-1331852 (BCL-xL inhibitor) have been described to affect ∆ψ_m_ in hematological malignancies accompanied by swollen mitochondria, rupture of the mitochondrial outer membrane as well as loss of cristae structure (Henz et al. 2019). As dysfunctional mitochondria may result in a drop of energy production (Auger et al. 2015), we checked the effect of GP on ATP production and loss of spare respiratory capacity (SRC, also called mitochondrial reserve capacity) being a sign for mitochondrial dysfunction (Marchetti et al. 2020). ATP production was significantly decreased in squamous SCL-1 cells compared to normal keratinocytes. Additionally, tumor cells showed no spare respiratory capacity (SRC) after 2.5 µM GP treatment in contrast to NHEK. In addition, we measured a higher proton leak in SCL-1 cells at a concentration of 1 µM GP compared to NHEK. This is in line with earlier published data indicating that gossypol has an uncoupling effect on rat liver mitochondria (Abou-Donia and Dieckert 1974) and TM4 cells originally derived from mouse testicular cells (Reyes et al. 1986).

In contrast to the finding in A375 melanoma cells (Haasler et al. 2021), SCL-1 carcinoma cells showed a different mechanism of cell death upon GP treatment. Although drastic effects on mitochondria were observed in SCL-1 cells, no apoptotic markers were found. Here, the necroptosis inhibitor necrostatin-1 resulted in a partial rescue of GP-induced cytotoxicity of the tumor cells, suggesting that necroptosis (Gong et al. 2019) might be involved. In that context, the BH3 mimetic drug obatoclax (GX15-070) also induced necroptosis in human oral squamous cell carcinoma (Sulkshane and Teni 2017). In addition, the necroptotic pathway shares morphological features of necrosis, including plasma membrane permeabilization/rupture and release of intracellular content such as lactate dehydrogenase (Gong et al. 2019), which we also found in this study. In the study of Maeda and coworkers, plasma membrane rupture was observed after mitochondrial fragmentation in the immortalized T lymphocyte cell line Jurkat (Maeda and Fadeel 2014) suggesting an interplay between mitochondrial effects and necroptosis. The switch from apoptosis to necroptosis usually depends on the activation status of caspase 8. Functional caspase 8 induces the extrinsic pathway of apoptotic cell death, whereas the inhibition of caspase 8 triggers the formation of necrosome and, thus, promotes necroptotic cell death (Fritsch et al. 2019). In line with this, no caspase 8 activity was observed in SCL-1 carcinoma cells suggesting that non-functional caspase 8 might be responsible for the switch of the cell death pathway. Recently, it was published that an interplay exists between autophagy and necroptosis, but the role of autophagy in necroptosis is still controversially discussed with regard to prevention or promotion of necroptosis (Zhang et al. 2022). As we do not see a full rescue from GP-initiated cytotoxic effect with the inhibitor necrostatin-1, we may speculate that autophagic processes might play a role in the GP-mediated cell death of the studied squamous carcinoma cells. Interestingly, it was demonstrated that (−) gossypol treatment resulted in an increased cytotoxicity on the lung carcinoma cell line A549 which is mediated by an upregulation of autophagic processes (Cai et al. 2022). Future studies will focus on that aspect.

In conclusion, our study showed for the first time, that, in addition to its main function as an inducer of apoptosis, (±) gossypol also promotes a necroptotic cell death in squamous skin cancer cells. The selectivity of the substance within a concentration range may also offer the possibility to use it for the effective treatment of squamous skin carcinoma in vivo without affecting normal (healthy) cells and, thus, alleviate harmful effects.

## Data Availability

The datasets generated during and/or analyzed during the current study are available from the corresponding author on reasonable request.
